# DNA assay based on Nanoceria as Fluorescence Quenchers (NanoCeracQ DNA assay)

**DOI:** 10.1038/s41598-018-20659-9

**Published:** 2018-02-05

**Authors:** Gonca Bülbül, Akhtar Hayat, Fatima Mustafa, Silvana Andreescu

**Affiliations:** Department of Chemistry and Biomolecular Science, Clarkson University, Potsdam, New York, 13699 United States

## Abstract

Functional nanomaterials with fluorescent or quenching abilities are important for the development of molecular probes for detection and studies of nucleic acids. Here, we describe a new class of molecular nanoprobes, the NanoCeracQ that uses nanoceria particles as a nanoquencher of fluorescent oligonucleotides for rapid and sensitive detection of DNA sequences and hybridization events. We show that nanoceria forms stable and reversible bionanoconjugates with oligonucleotides and can specifically recognize and detect DNA sequences in a single step. In absence of the target DNA, the nanoprobe produced minimal background fluorescence due to the high quenching efficiency of nanoceria. Competitive binding of the target induced a concentration dependent increase in the fluorescence signal due to hybridization and release of the fluorescent tag from the nanoparticle surface. The nanoprobe enabled sensitive detection of the complementary strand with a detection limit of 0.12 nM, using a single step procedure. The results show that biofunctionalized nanoceria can be used as a universal nanoquencher and nanosensing platform for fluorescent DNA detection and studies of nucleic acid interactions. This approach can find broad applications in molecular diagnostics, sensor development, gene expression profiling, imaging and forensic analysis.

## Introduction

The development of rapid and sensitive detection of nucleic acids is of significant scientific and biomedical importance for early diagnosis and screening of disease, to improve patient care, quality and outcomes in the healthcare system^1,2^. The discovery of nucleic acid biomarkers for many chronic diseases such as cancer^3,4^ and diabetes^5^ and the available genetic information of many diseases^6^ have generated an increased interest in biomolecular based diagnostics^7,8^. Consequently a variety of methods involving optical^9^, electrochemical^10^ and fluorescent^11^ transduction have been reported for the detection of nucleic acids. In the last decade, several types of nanomaterials have been explored to engineer functional nanoprobes for study of biomolecular interactions^7,12,13^ and design biodiagnostic tools for detection of nucleic acids and nucleic acid targets^9^.

To increase sensitivity and selectivity of nucleic acid detection, fluorescence based technologies that utilize fluorescent DNA probes have been developed. Some of the simplest fluorescence technologies rely on the fluorescence energy transfer resonance and use a fluorophore and its spectrally matched quencher. This “lights on” bind-and-detect fluorescence quenching assays has made them ideal tools for point-of-care measurements in environments with limited resources or access to laboratory facilities. Portable handheld fluorometers such as the QuantiFluor™ (Promega Corp.) have been developed to address the need of instrumentation for point-of-care measurements using devices that are simple, easy to handle, cost effective and suitable for on-site analysis. DNA-based assays with one step detection capabilities via fluorescence quenching have found a great deal of interest in recent years for numerous applications^14,15^. These methods showed increased convenience in analysis, rapid hybridization kinetics and sensitivity^16,17^. The use of fluorescent DNA probes usually involves the design of a fluorescent probe-quencher pair and labelling of oligonucleotides with fluorescent dyes to transduce the molecular recognition event to measurable fluorescence readout^18^. Fluorescence energy transfer of this pair in the presence of the target strand is then related to the DNA hybridization events monitored by the change in the fluorescence intensity. Traditionally, these methods required the use organic quenchers to quantify hybridization events. More recently, conventional organic quenchers were replaced with nanoquenchers such as gold nanoparticles^19^, carbon nanotubes^20^ and graphene^21^. The advantages of these materials include high stability, increased quenching, and the capability to enhance selectivity by using several fluorophores simultaneously^20,22^. Despite significant progress in the use of nanomaterials for nucleic acid detection, there exist several challenges related to the interface between the nanomaterial surface and the DNA oligonucleotides, in particular the ability to precisely control assembly and fluorescence emission properties^23^. Other challenges include background interferences, surface effects and salts induced aggregation, which limit their detection capability. To be able to generate nucleic acid assays that are easy-to-use, stable and robust, there is a need to design new materials and methods that provide better control over the interfacial properties and nanoassembly of the DNA probe to the nanomaterial surface.

We herein describe a new class of fluorescence quenchers for the detection of nucleic acids using nanoceria particles. These particles have unique and unusual physicochemical properties at biological interfaces that are substantially different from other nano-based quenchers. First, their surface properties provide reversible and controllable assembly of fluorescent DNA probes with fast adsorption and desorption capacity without base modification. Second, nanoceria serves as a general biocompatible substrate for reversible attachment of nucleic acids due to the strong interaction between cerium and the phosphate backbone^24^. Third, these particles generate strong target-induced fluorescent changes, enhancing sensitivity of analysis. In this work, we explore the use of nanoceria as a robust nanoplatform for nucleic acid detection and study of nucleic acid interactions and hybridization. Nanoceria particles have gained increased interest in the last decade due to their powerful catalytic and optical properties^25^ which enabled novel applications in bioanalysis. In previous studies, nanoceria has been used as a bio-immobilization support, colorimetric probe and signal amplifier in the design of enzymatic biosensors^26,27^ and as a replacement of enzyme labels in aptamer assays with colorimetric^24^ and electrochemical^28^ detection. Similarly, fluorescence quenching properties of nanoceria particles have been explored for various analytical applications such as adsorption, discrimination and detection of anions^29–31^. In the same context, Liu *et al*. reported the use of nanoceria for detection of H_2_O_2_ and study of heavy metal adsorption using DNA, but those studies involved a competitive multistep mechanism and were not designed for DNA measurements. In this study, we demonstrate the use of nanoceria as a fluorescent dye quencher pair for sequence-specific detection of DNA hybridization reactions. To the best of our knowledge, this is the first report on the use of nanoceria particles as functional nanoprobes for sensitive DNA analysis in one-step. This novel platform is sensitive and selective, and provides a versatile platform for study of DNA interactions. This approach may find applications in diagnostics, forensic analysis, imaging and environmental monitoring.

## Results and Discussion

### Detection Method – Fluorescence Quenching and Hybridization Test

**Figure 1:**
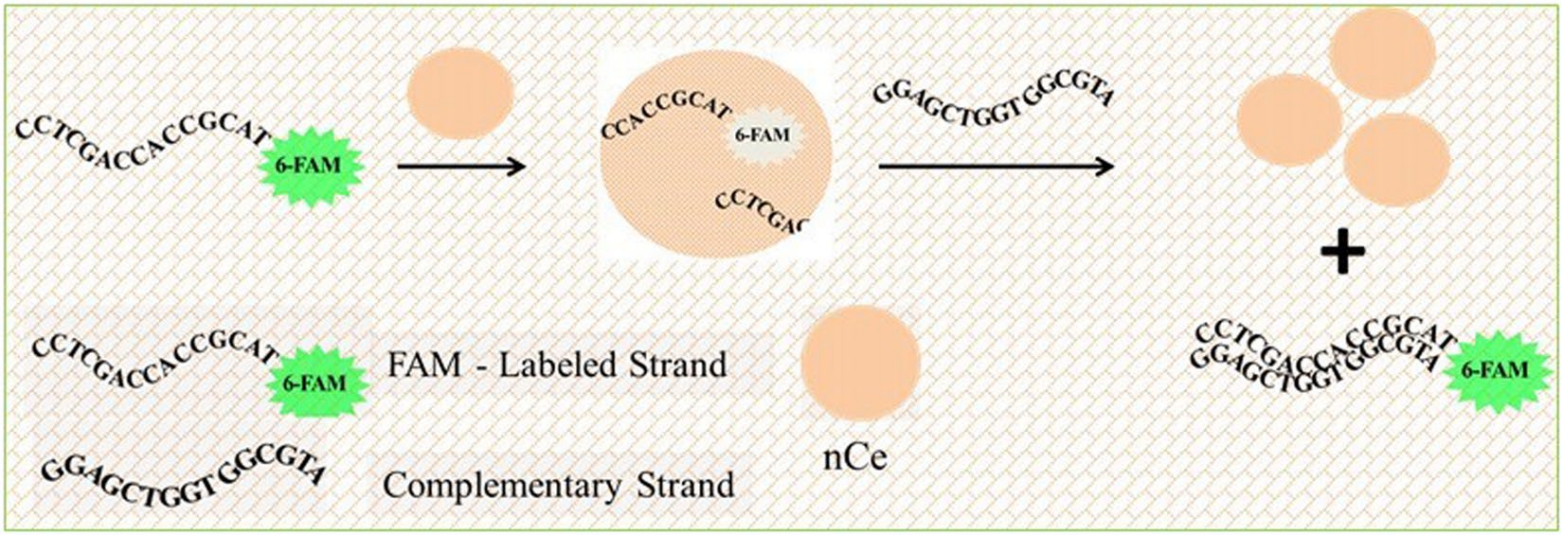
Schematic representation of the detection mechanism showing target hybridization to nanoceria conjugated FAM-ssDNA. Upon hybridization, the fluorescent FAM probe is released from the particle surface, restoring the fluorescent signal.

To design the fluorescence assay, we first developed the nanoceria-based probe by modifying the surface of nanoceria particles with a fluorophore (FAM)-labelled ssDNA. Earlier studies have shown that ssDNA has strong affinity for nanoceria surfaces by a mechanism involving the interaction between the phosphate backbone and cerium at the particle surface^24,32,33^. In previous work, binding between nanoceria and DNA was demonstrated with several chain lengths including short and long oligonulecotide strands of 15, 36 and 77 mers^24^. The attachment of DNA on nanoceria surface involves electrostatic interaction and specific binding through the phosphate backbone of the DNA. These interactions facilitate strong and reversible assembly in a single step, without prior modification. In this work, we used a FAM-labelled ssDNA to generate the nanoprobe. Commercially available cerium oxide nanoparticles with an average size of ~28 nm were used in this study. TEM images and dynamic light scattering indicate an average size of ~28 nm (Figure S1, Supplementary information (SI)). These particles are positively charged with a zeta potential value of +38.58 mV (HEPES buffer; pH 7.4), which facilitates electrostatic assembly to the negatively charged ssDNA. The zeta potential of the DNA-bound nanoceria decreased to +10 mV, indicating changes in the nanoparticle surface. The decrease in the positive charge of nanoceria indicates the attachment of FAM labelled DNA. Lack of complete change to a neutral/negative nanoceria surface upon attachment of DNA is due to the highly positive surface of nanoceria which is predominant and strong enough to compensate the negative charge of DNA; however the significant decrease in the positive charge at the surface of the DNA-nanoceria complex shows a clear change in surface properties due to DNA attachment. Figure 1 illustrates the detection mechanism of the proposed fluorescent assay for nucleic acid detection. The strong binding affinity of nanoceria for the FAM-ssDNA is expected to quench the fluorescence of the FAM label, suppressing the fluorescent signal. In the presence of target, the formation of dsDNA duplexes upon hybridization which occurs concurrently with a conformational transition of the surface adsorbed ssDNA probe from random coil to a rigid structure is expected to induce desorption of the probe from the nanoparticle surface. Surface displacement can result in recovery of the fluorescence signal, directly proportional to the amount of target analyte.

**Figure 2:**
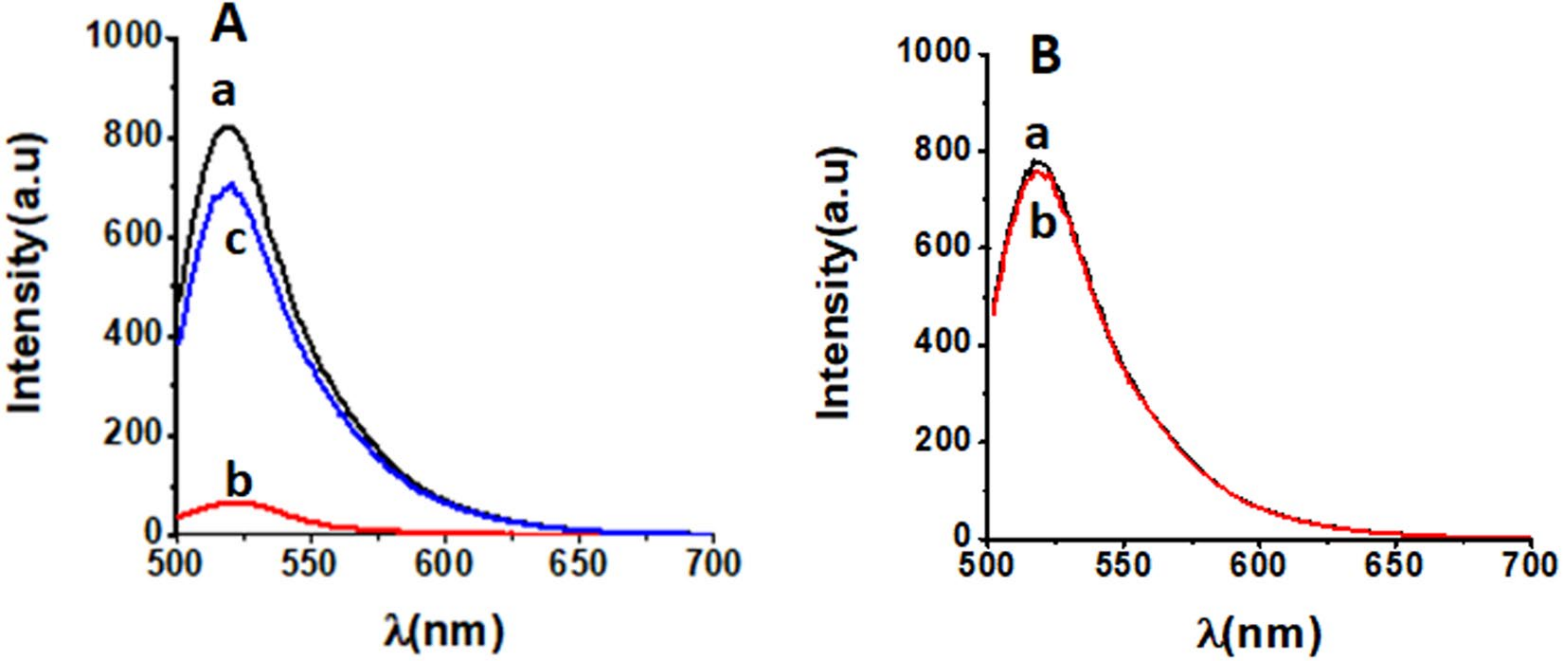
Fluorescence spectra of FAM-labeled ssDNA strand: (**A**) in the absence (a) and presence of 2.2 mg/L nanoceria (b), and after addition of complementary strand (c). (**B**) Control in absence of the nanoceria quencher with (a) and without 37 nM complementary strand (b).

To confirm the detection mechanism, we tested the fluorescent properties of the FAM-ssDNA and the nanoceria-FAM-ssDNA before and after addition of the complementary DNA target. As expected, the FAM labeled ssDNA strand exhibits high fluorescence signal. The fluorescence intensity of the FAM-ssDNA conjugated to nanoceria is quenched significantly, resulting in a very low signal. This result provides evidence of the fluorescence quenching of the FAM tag by nanoceria. The quenching mechanism of CeO_2_-FAM system can be attributed to the electron transfer between excited fluorophores and nanoceria^32,34^. The nanoceria has large band gap with semiconducting properties. The electrostatic interaction involving Ce-O bond between CeO_2_ and FAM can result in fluorescence quenching through the acceptance of electrons from excited FAM molecules. Upon addition of the complementary target, a dramatic increase in the fluorescence intensity was observed suggesting that the FAM labeled ssDNA was released from the nanoceria surface. The increase in the signal was concentration dependent, which enabled the development of a functional nanoprobe for detection of DNA (Fig. 2A). To further show that quenching is a result of nanoceria, a control experiment with the FAM-labeled and complementary strand, in the absence of particles, was performed. In the absence of the nanoceria quencher there was no change in the fluorescence signal further demonstrating the detection mechanism (Fig. 2B). The essential difference arises because ssDNA can uncoil sufficiently to expose its bases, whereas dsDNA has a stable double-helix geometry. Although nanoceria had a positive charge after attachment of the FAM ssDNA, the difference in electrostatic properties and the relatively less exposed bases in the duplex DNA, along with the strong binding affinity between the two complementary strands can drive the detachment of strands from the nanoceria surface and subsequently the recovery of fluorescence response.

We further evaluated whether the interaction of DNA with nanoceria and/or target is affecting the DNA structure using Circular Dichroism Circular dichroism (CD)^35,36^. Figure S2 shows ellipticity curves for nanoceria, FAM-ssDNA, FAM-ssDNA in the presence of nanoceria and FAM-ssDNA strand in the presence of both nanoceria and CS (Figure S2). The FAM-DNA strand attached to nanoceria displays a similar CD/mdeg ellipticity spectra to that of FAM-DNA only, indicating similar supramolecular organization. However upon incubation with the CS strand, we observe a change in the maxima of the CD/mdeg spectra at a slightly different wavelength, which indicates conformational changes due to duplex formation.

### Optimization of Operational Parameters

To optimize the assay and design the nanoceria-based fluorescent quencher probe, NanoCeracQ, we first investigated the interaction and binding mechanism of the ssDNA to the surface of nanoceria. Experiments were performed in HEPES buffer at a pH of 7.4 with a concentration of 8.3 nM FAM-labeled-ssDNA which provided the higher intensity for the fluorescently modified FAM strand (Figure S3 in SI). Nanoceria is positively charged at pHs lower than ~8. Therefore, charge repulsion with ssDNA can be expected at pH values greater than 8. However, in earlier studies it has been shown that DNA can still adsorb on the surface of nanoceria at high pH values. This behavior was attributed to the specific binding affinity of DNA through the DNA phosphate backbone, in addition to the electrostatic interaction^32^. Therefore, in this work we tested the quenching efficiency of nanoceria at different pH values. Interestingly, nearly 100% of the fluorescence intensity of FAM-modified ssDNA was quenched by nanoceria at all pHs tested, showing that nanoceria exhibits high fluorescence quenching ability as well as a strong affinity for ssDNA (Figure S4). A physiological pH of 7.4 was selected for subsequent experiments.

**Figure 3:**
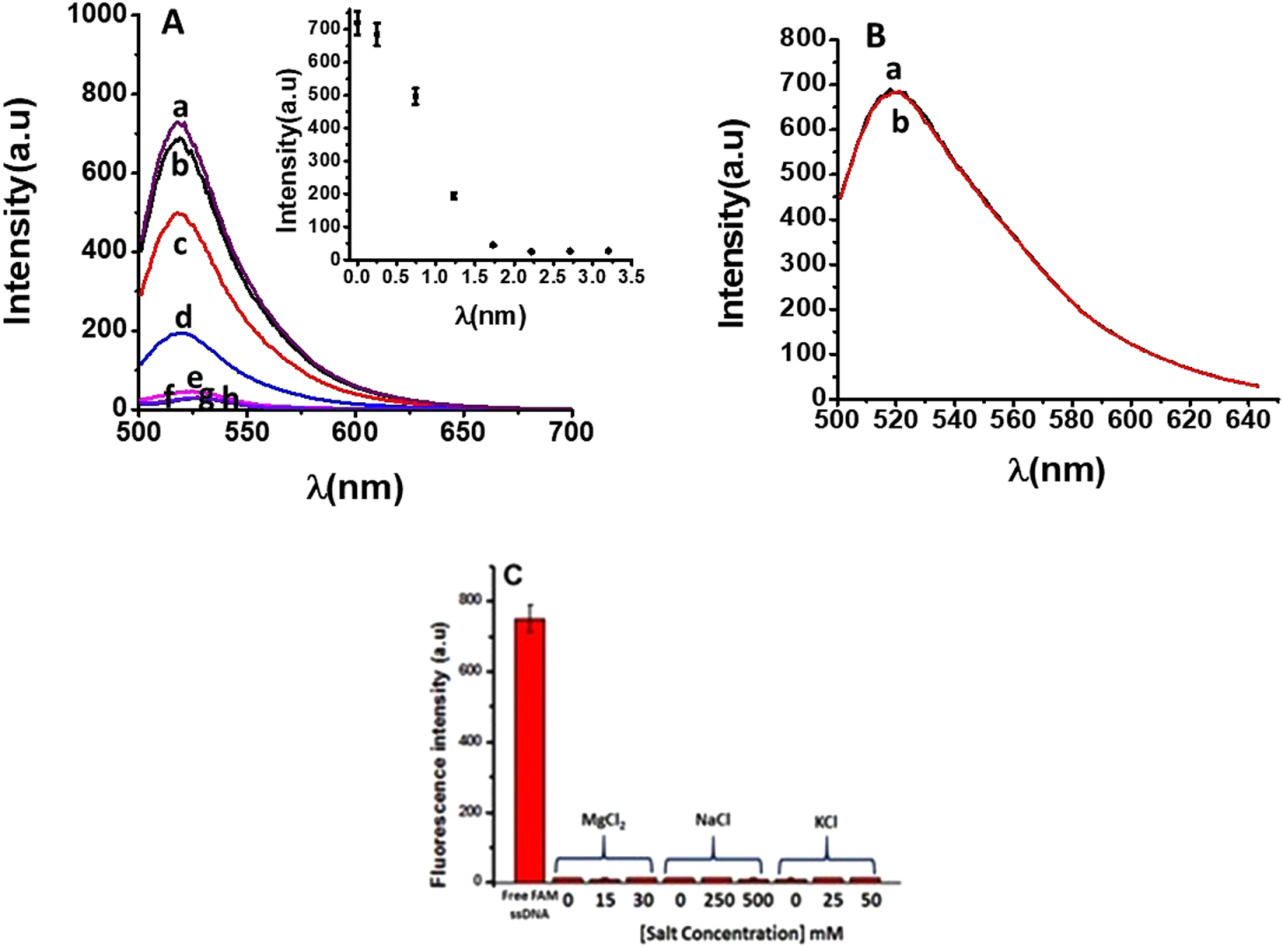
Fluorescence spectra of FAM-labeled DNA strand (8.3 nM) (**A**) in the presence of different concentrations of nanoceria particles (ppm); (a) 0, (b) 0.25, (c) 0.75, (d) 1.25, (e) 1.75, (f) 2.2, (g) 2.75, (h) 3.2; while inset shows the particle concentration versus fluorescence quenching (**B**) in the presence of silica nanoparticles, a) 0, b) 2.2 mg/L. (**C**) Effect of salt concentrations on the adsorption of FAM-ssDNA on nanoceria; the fluorescence response of the free FAM-ssDNA was considered as a reference while the amount of adsorbed DNA was estimated based on the quenched fluorescence response by nanoceria (2.2 mg/L).

The amount of particles and their available surface determine the adsorption and self-assembly of the FAM-ssDNA as well as the quenching efficiency. As shown in Fig. 3A, the intensity of the fluorescence response decreases with increasing concentration of nanoceria. For particle concentrations up to 3.7 mg/l, 96% of the fluorescence intensity of FAM-labeled ssDNA was quenched. Concentrations higher than 3.7 mg/L caused an increase in the baseline fluorescence. Based on this result, the analytical characterization was performed using a concentration of 3.7 mg/L nanoceria. Next we investigated whether the observed effects are a consequence of the nanosized effect of these particles. For comparison, three different types of commercially available ceria particles were investigated with diameters of (nm) 28.1 ± 5.62, 450 ± 25, 800 ± 50 based on particle size distribution measurements (Figure S5). Only the nanosized particles exhibited well defined quenching, while the larger particles did not show any significant change in the fluorescence signal. The decrease in fluorescence quenching as compared to the well dispersed particles can be related to a decrease in surface area due to precipitation or agglomeration of the nanoparticles. Although the fluorescence quenching is a common feature of nanoceria, the extent of quenching varies with the particle size and agglomeration status. For comparison, experiments were also performed with silica nanoparticles with a surface charge of −30.7 mV. Figure 3B shows no quenching of the FAM-labeled DNA by silica, which indicates no binding of DNA to these particles. These results demonstrate that the ceria surface is responsible for DNA binding. Therefore, following experiments were performed with the 28 nm nanoceria particles. Further experiments were performed to understand the impact of salt concentration on the DNA adsorption process. The fluorescence response of the free FAM-ssDNA was used as a reference to investigate the extent of ssDNA adsorption on nanoceria. The amount of adsorbed DNA can be estimated based on the quenched fluorescence response by nanoceria. Figure 3C shows the effect of salt concentration. No effect on the DNA adsorption on nanoceria was observed for salt concentrations of up to 30 mM MgCl_2_, 500 mM NaCl and 50 mM KCl (Fig. 3C). Higher concentrations of salts induced the precipitation of particles; however, such high salt conditions are not typically present in biological media.

### Kinetics of Nanoceria Quenching Efficiency

The kinetic and the DNA binding behavior of biomolecules to nanoceria are determined by the specific binding interactions to the nanoceria surface. Adsorption of ssDNA on nanoceria is a rapid process. Figure S6 shows fluorescence quenching of FAM-labeled ssDNA by nanoceria as a function of time. The quenching kinetics was fast, with nearly 100% fluorescence quenched in less than 1 minute. We also note that quenching also takes place with conventional fluorescence dye such as rhodamine 6G in aqueous media (Figure S7). We believe that the nanoceria surface exhibiting both charge attraction with the negatively charged ssDNA and specific cerium-phosphate binding affinity is the main contributor to the fast adsorption rate. This characteristic is unique to nanoceria and it is what sets this material apart from other nanomaterial quenchers such as gold nanoparticles or carbon nanotubes. Therefore, surface modification of nanoceria is a straightforward process which adds convenience in analysis. We highlight that in the case of other materials such as gold nanoparticles and carbon-based nanomaterials attachment is based primarily on charge attraction with negative charged ssDNA (electrostatic interaction) and those lack affinity for phosphate. Since the attachment of DNA on nanoceria surface takes place through two phenomena including electrostatic interaction with possible contribution from the hydrogen bonding and the affinity of phosphate for cerium, this can possibly enhance the DNA adsorption capacity of the nanoceria as compared to that of other materials. The increased adsorption of FAM-ssDNA can result in better fluorescence quenching properties. In other words, we can say that a relatively low concentration of nanoceria would be required in comparison to that of other materials to have similar adsorption of DNA for the same extent of fluorescence quenching. Moreover, as nanoceria is known to have exchangeable ^+^3/^+^4 oxidation states, and nanoflourescence quenching involves transfer of electrons, the oxidation state at its surface further enhances the fluorescence quenching.

When the FAM labeled strand was hybridized with its complementary strand to form a duplex, the FAM fluorescence was fully recovered. This shows that the interaction between ssDNA and nanoceria is reversible and relatively weak as compared to the interaction between the complementary strand and ssDNA, suggesting different adsorption behavior of nanoceria for ssDNA as compared to dsDNA. Since ssDNA binds to nanoceria in a noncovalent manner, this can be released from its surface competitively by sequence-specific hybridization. Competitive binding is due to the stronger affinity of the complementary strand for the fluorophore labeled ssDNA. Presence of the complementary strand in the system resulted in hybridization and release of the fluorophore-labeled strand, consequently increasing the fluorescence signal.

### Target Detection and Selectivity - Analytical Performance Characteristics

**Figure 4:**
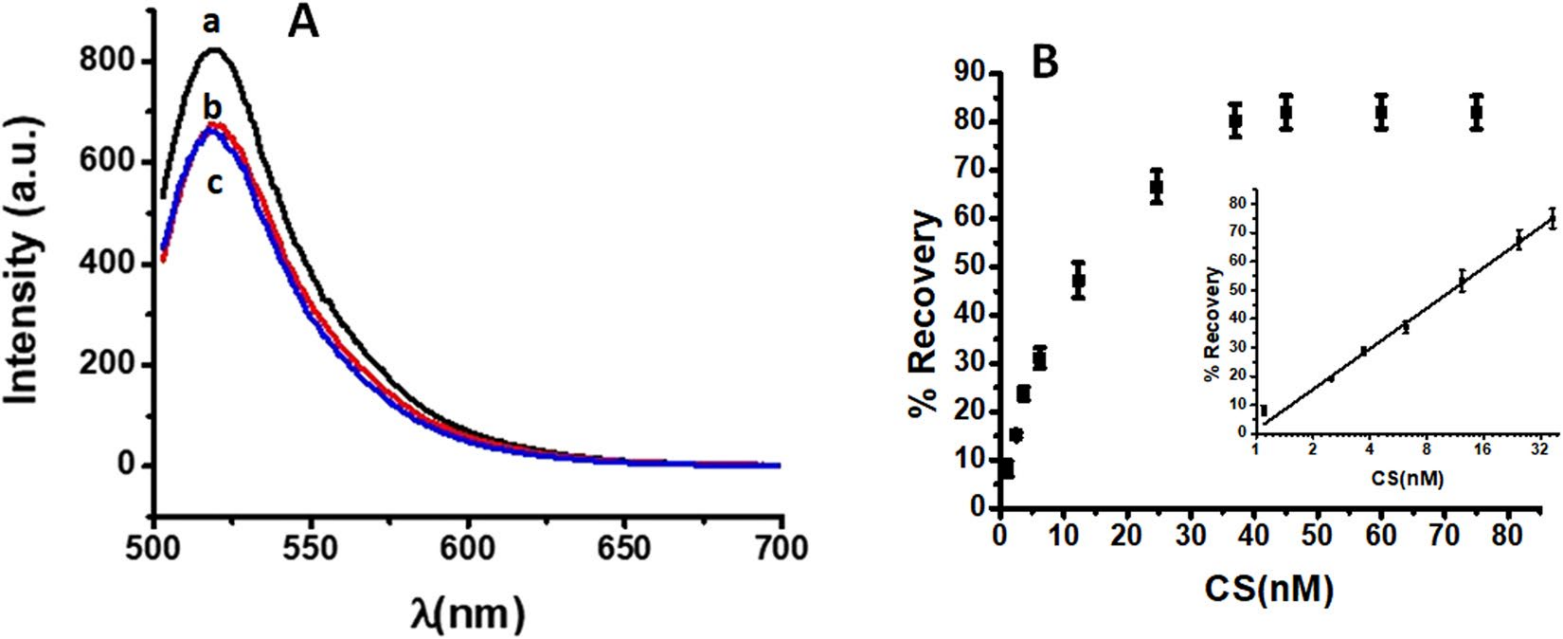
(**A**) Calibration curve obtained after addition of different concentrations of the complementary strand in the presence of nanoceria particles. Error bars represent the standard deviations for n = 3 replicates. (**B**) Effect of incubation time for hybridization of FAM labeled strand (8.3 nM) in the presence of CS (30 nM), (a) 0 min, (b) 5 min.

The self-assembled FAM-ssDNA nanoceria was further used as an analytical probe for detection of the complementary DNA target. Using the optimized conditions and employing a fixed concentration of nanoceria probe, a gradual increase in the fluorescence response was obtained upon addition of the complementary strand (Fig. 4). The fluorescence response is linearly related to target concentration in the range 1.1 to 37 nM. On the basis of control experiments with buffer, we have calculated a detection limit of 0.12 nM. The response is almost instantaneous (Fig. 4B) and analysis with this assay is a single step process involving both hybridization and quenching with liberation of the dye. The measurements showed good reproducibility with a coefficient of variation below 5%. This method compares favorably with other nanoparticle quenchers, summarized in Table S2. As compared to other particle systems, the interaction between nanoceria and DNA is a unique property of these particles due to the specific binding between cerium and the phosphate groups in nucleic acids and in principle can be applied to other DNA molecules.

As it can be seen from Fig. 4, when the concentration of the complementary strand was increased, more ssDNA were released to the solution resulting in increase in the fluorescence response. Based on this phenomenon, we have used the equation below to calculate the percentage recovery.

$$ \% \,{\rm{Fluorescent}}\,{\rm{Recovery}}=({{\rm{F}}}_{{\rm{t}}}-{{\rm{F}}}_{{\rm{b}}})/({{\rm{F}}}_{{\rm{c}}}-{{\rm{F}}}_{{\rm{b}}})\times 100$$

Where: the F_b_ is the fluorescence response of the buffer, F_c_ is the fluorescence response of the FAM-ssDNA containing buffer medium and F_t_ is the fluorescence response observed in the presence of a given concentration of complementary ssDNA strand.

**Figure 5:**
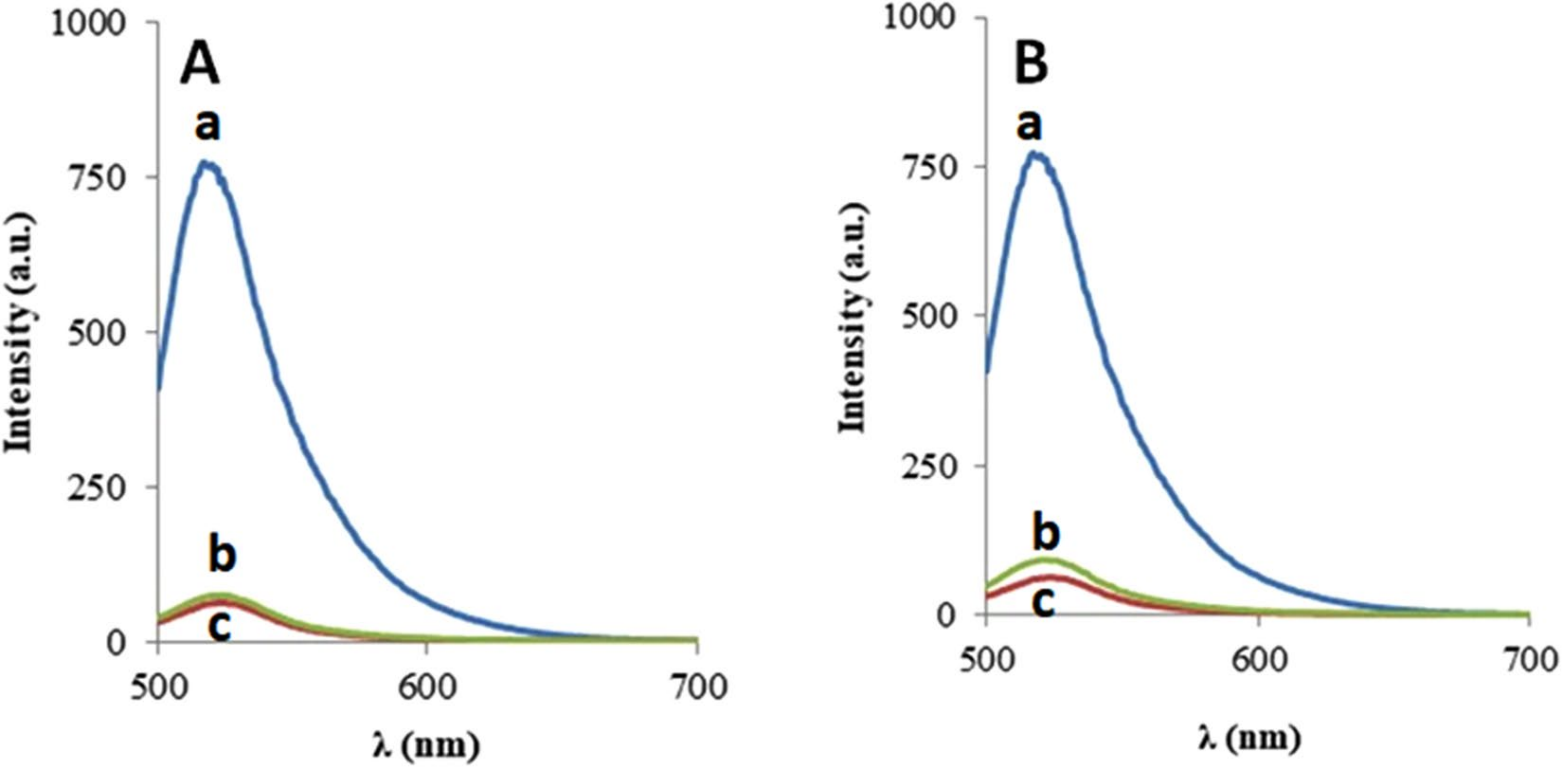
Fluorescence spectra of FAM labeled strand in the absence (a) and presence of nanoceria (c), and after addition of 24.6 nM interfering strands (b) showing almost no change in the fluorescence signal for**:** IS1 in (**A**) and IS2 in (**B**). (n = 3; SD/σ =  ± 8).

To determine the specificity of analysis towards the detection target, a control experiment was performed using two different strands with similar lengths. As can be seen from the Fig. 5 and Table S1, the strand IS2 with a single mismatch to FAM-ssDNA did not recover the fluorescence response, indicating that a complete pairing base is needed to recover the fluorescence response. The absence of fluorescence changes after addition of these strands indicate excellent ability to discriminate against the two non-complementary sequences.

**Figure 6:**
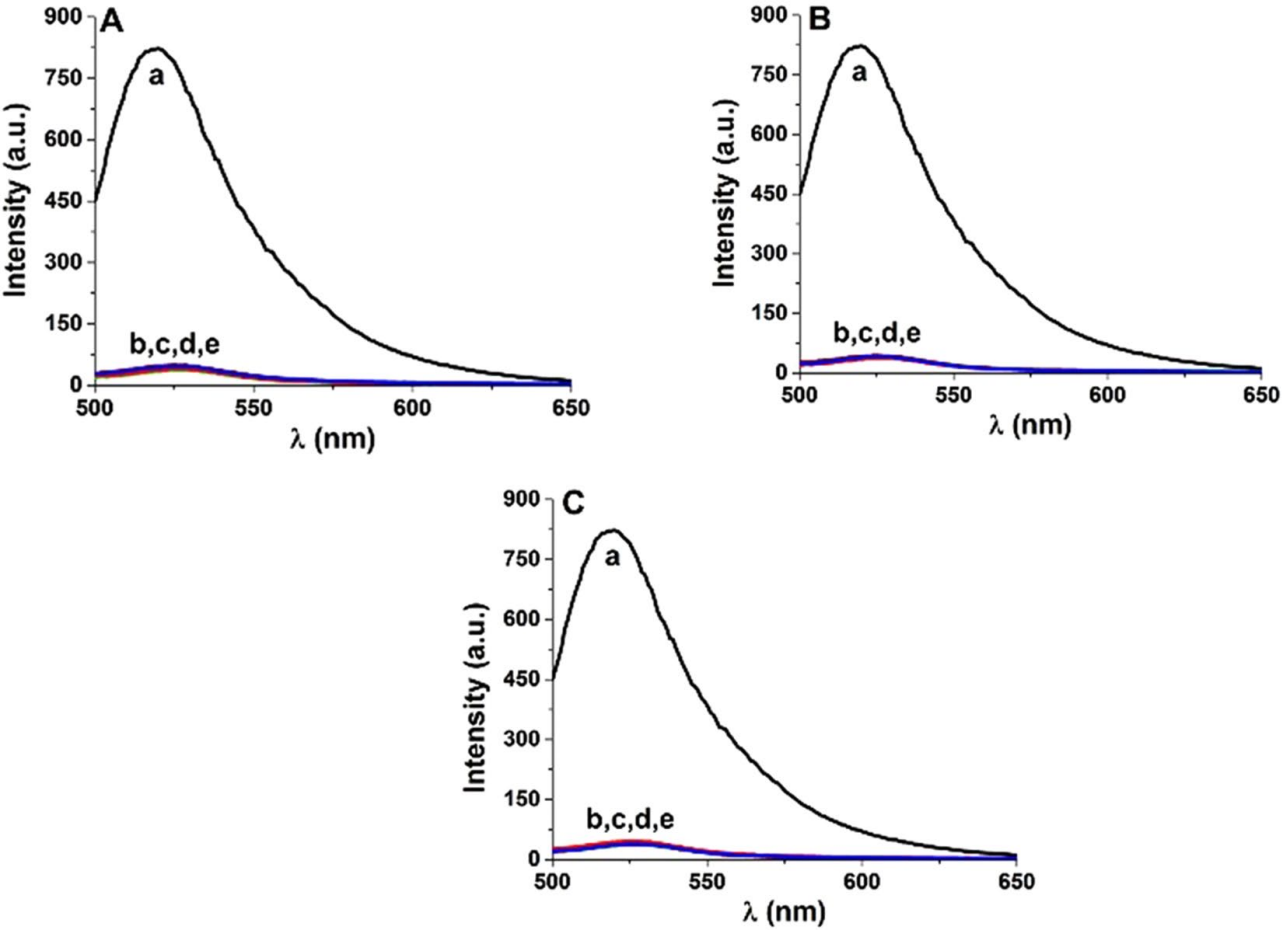
Fluorescence spectra of FAM-labeled ssDNA strand of 8.3 nM in the absence (a) and presence of nanoceria (e), and after addition of complementary strand (nM); (b) 24.6, (c) 12.3, (d) 6.15: (**A**) IS3, (**B**) IS4, (**C**) IS5.

Experiments with varying concentrations of complementary DNA strands shorter than the FAM ssDNA were performed to study the effect of strand length. The nucleotide sequences are listed in Table S1. The fluorescence responses obtained in the absence and presence of these strands and control experiments are shown in Fig. 6. The shorter strands did not recover the fluorescence response. These experiments further confirm that a complete base pair match is required for fluorescence recovery and indicate that the complementary strands must fully detach the FAM strands from the nanoceria surface to recover the fluorescence response. These experiments show the ability of this methodology to differentiate single mismatch in a given complementary strand. The fluorescence recovery takes place only in case of complete detachment of FAM-ssDNA from nanoceria surface. The fluorescence quenching/recovery phenomena depends on the extent of DNA adsorption, nanomaterial quenching efficiency and the ratio between binding affinities of FAM DNA-CS and FAM DNA-nCe. The two phenomena including electrostatic interaction with possible contribution from the hydrogen bonding and the affinity of phosphate for cerium makes relatively stronger attachment of DNA on nanoceria surface as compared to that of other materials. It can be assumed that the binding energy of single base mismatch CS with FAM-DNA was not enough to compensate/overcome the strong binding/attachment affinity between FAM DNA and nCe, to detach the FAM from nCe surface. A similar effect on fluorescence recovery was observed when shorter complementary strands IS3, IS4 and IS5 were employed (Fig. 6).

The DNA assay described here based on direct quenching of a nanoceria probe has several advantages over other nanomaterial-based fluorescence quenching assays. The method does not require a long incubation time or complicated labeling steps as compared to other methods^37–39^; a complete test requires approximately 1 min with almost 100% fluorescence quenching efficiency and a 90% recovery of the fluorescence response within a fraction of time. A variety of nanoceria particles are available and can be used in this assay. The method is fast and involves minimum steps and reagent use and can be adapted in the future as a low cost test for point of care analysis.

## Conclusions

In summary, we have developed a novel nanoprobe for sensitive fluorescent analysis of DNA using self-assembled bioconjugated nanoceria particles. We demonstrated that nanoceria possesses high fluorescence quenching ability against FAM-labeled ssDNA, while also serving as an effective immobilization scaffold enabling reversible DNA binding, specific recognition and signaling of DNA/target hybridization events. The bioconjugated nanoceria complex represents a new class of fluorescence quenchers that offers several advantages over other nanoparticle quenchers for studying nucleic acid interactions. First, the preparation of the probe is straightforward and the measurement process is a single step procedure. These particles can be easily obtained in large quantities and are relatively inexpensive. Second, the quenching by nanoceria follows fast kinetics with nearly all fluorescence quenched within several seconds. Further, the release of the dye-labeled strand from the nanoceria surface upon hybridization and detection of the complementary strand is a fast process and the entire analysis can be completed within minutes, significantly simplifying analysis and reducing the number of steps. Third, the nanoceria surface provides an ideal chemistry for the attachment of DNA due to the binding of ceria to the phosphate backbone, offering a simple approach for the preparation of the probe. There is no need for the modification of the nanoparticle or of the fluorescently-labeled strand. Since the interaction between the nanoceria and DNA is generally applicable, the method may be extended to other oligonucleotides and the detection mechanism can be used as a universal platform for DNA detection and study of nucleic acid interactions. The ceria nanoprobe and the method reported here provide a new concept for the design of new devices for biomedical diagnosis, molecular sensing, forensics analysis, gene expression profiling, and imaging.

## Experimental Section

### Materials and Reagents

Cerium (IV) oxide 20% in H_2_O was from Alfa Aesar. Cerium oxide (IV)-20-gadolinium doped nanopowder and cerium oxide (IV)-15-samaria doped nanopowder, were from Sigma Aldrich. The FAM labeled DNA strand, its complementary strand (CS) and the other DNA strands (CI) were provided by Integrated DNA Technologies. Experiments to study the effect of strand length were performed with complementary DNA strands of varying lengths from Integrated DNA Technologies. The sequences are provided in Table S1. 10 mM HEPES buffer in the presence of 1 mM MgCl_2_, 140 mM NaCl, and 2.7 mM KCl was prepared.

### Experimental Procedures

Fluorescence measurements were performed with Varian Cary Eclipse Fluorescence Spectrophotometer. All reagents were used as received and all solutions were prepared using distilled and deionized water (Millipore Direct-Q- system) with a resistivity of 18.2 M ohms. A Zeta PALS from Brookhaven Instruments was used to determine the hydrodynamic diameter of the particles. High resolution transmission electron microscopy (HR-TEM) images were taken using a high-resolution JEOL 2010 transmission electron microscope.

### Fluorescence Assay - Characterization and Detection Procedure

All DNA strands were subjected to a preheat treatment before use. The DNA solution was heated at 95 °C for a time period of 5 min and subsequently left at room temperature for 3 min. Transmission electron microscopy (TEM), particle size distribution, and fluorescence experiments were performed to characterize the nanoparticles and their interaction with ssDNA. For performing quantitative analysis, solutions of the complementary strand in the concentration range of 1.1 nM to 37 nM were added to the FAM-labeled strand conjugated to nanoceria. Measurements were performed in HEPES buffer containing 1 mM MgCl_2_, 140 mM NaCl, and 2.7 mM KCl. Calibration curves were plotted from the fluorescence spectra (500–700 nm) before and after addition of complementary strand. Specificity and selectivity of the assay were determined by measuring the response of two different ssDNA probes (other than the complementary strand) with the ssDNA modified nanoceria probe. All experiments were performed in triplicate. Results of calibration curve and effect of experimental variables are shown with standard deviation for n = 3 independently run measurements.

### Data availability statement

All data generated or analyzed during this study are included in this published article (and its Supplementary Information files).

## Electronic supplementary material

Supplementary Information
