# Supplementary material for: DNA assay based on Nanoceria as Fluorescence Quenchers (NanoCeracQ DNA assay)
Source: Sci Rep. 2018 Feb 5;8:2426. doi: 10.1038/s41598-018-20659-9 (PMC5799284; doi:10.1038/s41598-018-20659-9)
Supplement: Supplementary file 1 — Supplementary Information [file 41598_2018_20659_MOESM1_ESM.pdf]

DNA assay based on Nanoceria as Fluorescence Quenchers (NanoCeracQ DNA assay)

*Gonca Bülbül, Akhtar Hayat, Fatima Mustafa and Silvana Andreescu**

Department of Chemistry and Biomolecular Science, Clarkson University, Potsdam, New York 13699, United States

Correspondence to; eandrees@clarkson.edu

Supplementary Information

Table S1: Oligonucleotide sequences used in this work.

	Sequence (5'→ 3')
FAM-labeled strand	TAC-GCC-ACC-AGC-TCC
CS	GGA-GCT-GGT-GGC-GTA
IS1	GGA-GCT-GGT-GGT-GGC-GTA
IS2	AGA-GCT-GGT-GGC-GTA
IS3	GCT-GGT-GGC-GTA
IS4	GGT-GGC-GTA
IS5	GGC-GTA

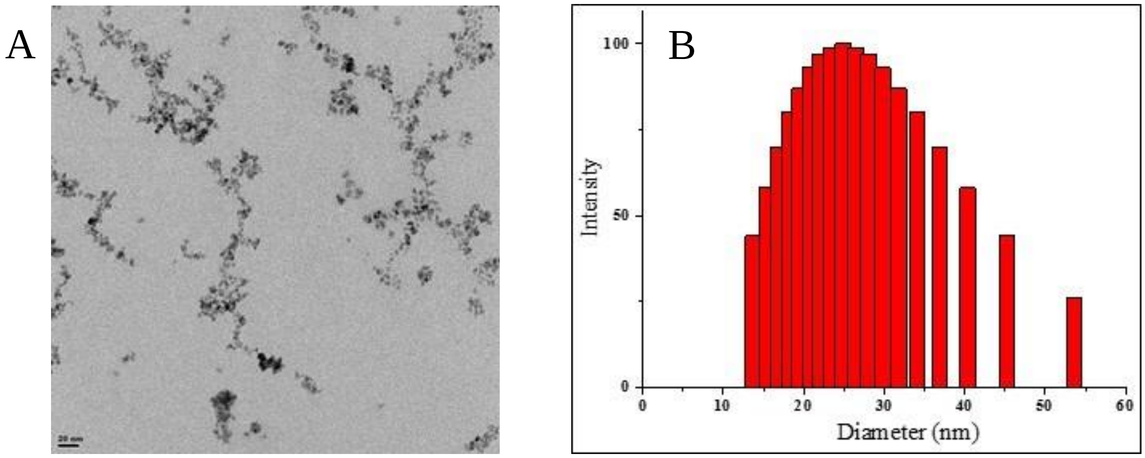

Figure S1. TEM image (A) and DLS diameter (B) of the nanoceria particles used in present study. The scale bar in (A) is 20 nm.

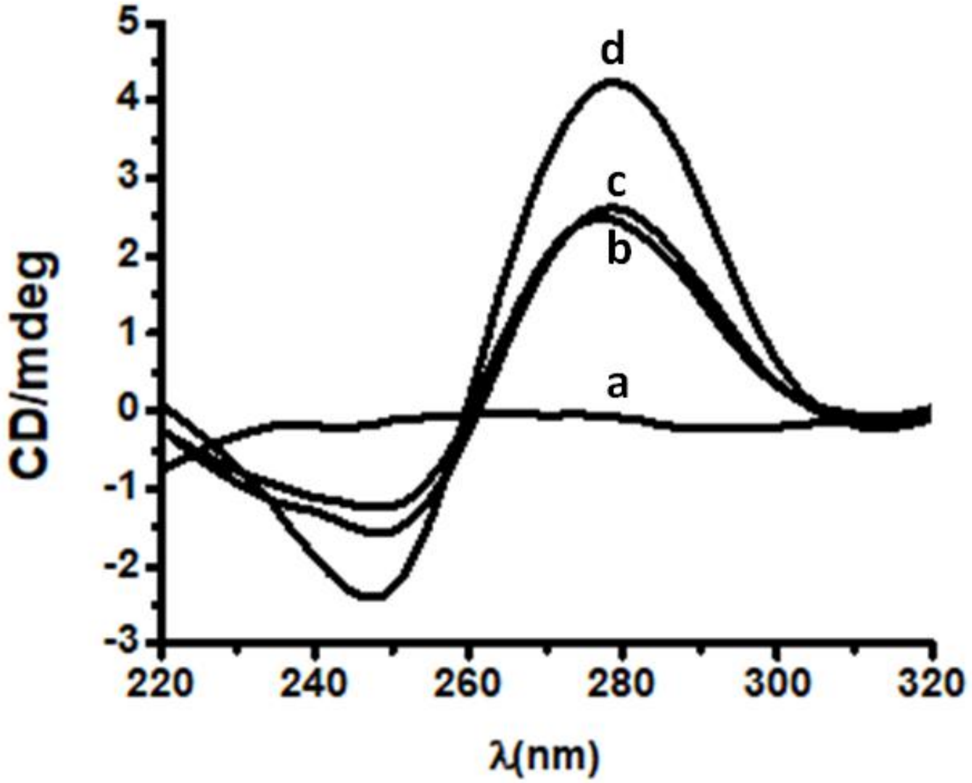

Figure S2. CD/mdeg profile of nanoceria (2 mg/L) a), FAM-ssDNA strand (10 µM) b), nanoceria (2 mg/L) + FAM-ssDNA strand (10 µM) c) nanoceria (2 mg/L) + FAM-ssDNA strand (10 µM) + FAM CS strand (10 µM)

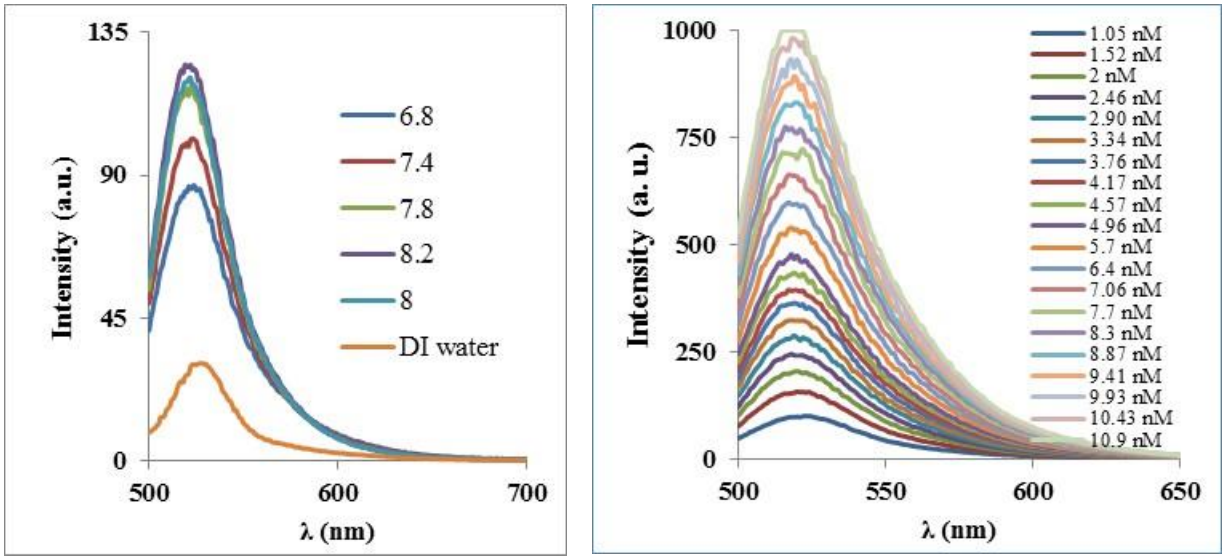

Figure S3. A) Fluorescence spectra of 1.05 nM FAM modified strand in 10 mM Hepes buffer in different pHs and in distilled water. **B)** Different concentrations of FAM-labeled strand in 10 mM Hepes at pH 7.4.

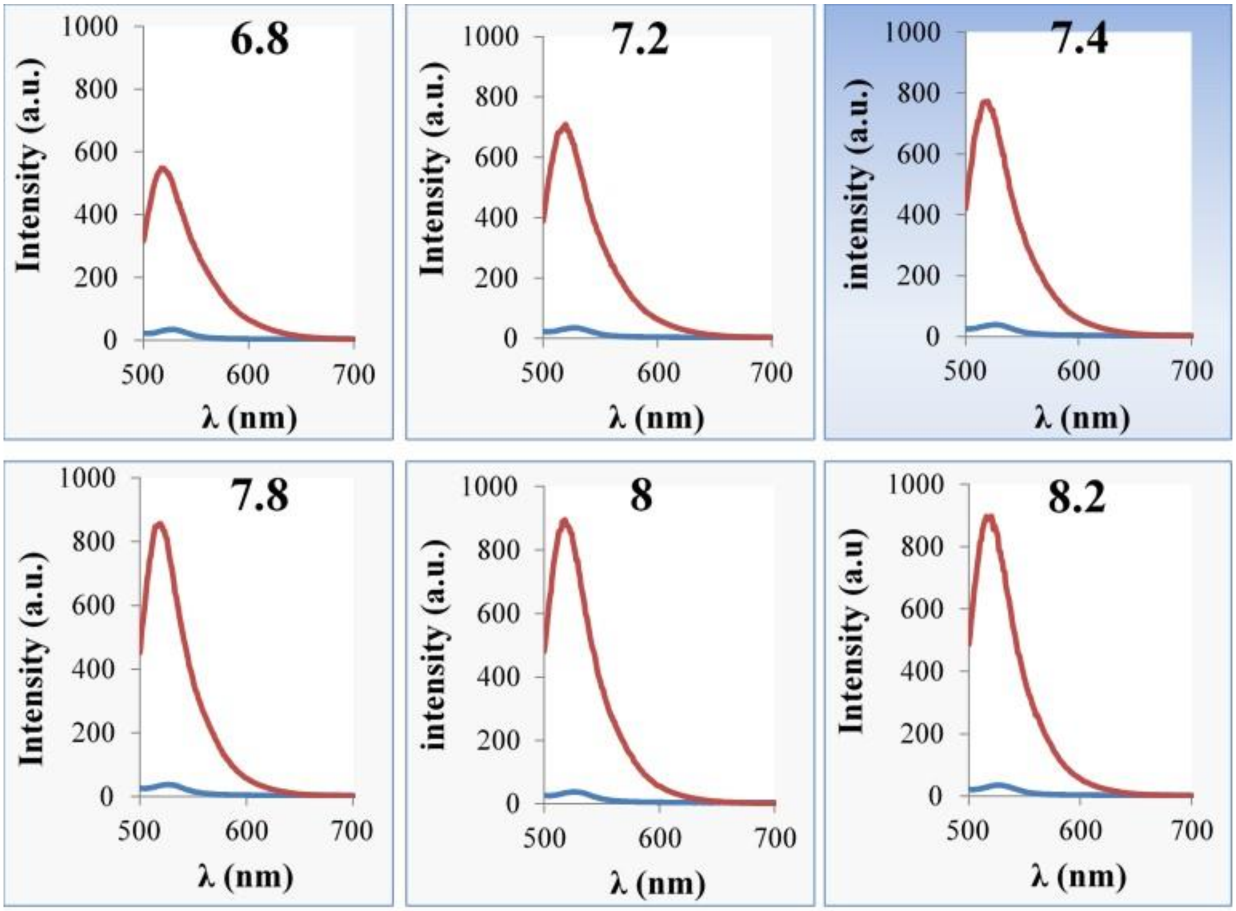

Figure S4. Fluorescence spectra of FAM-labeled ssDNA strand in the absence (red) and presence of 2.2 mg/L nanoceria (blue) at different pH values. Cerium (IV) oxide 20% in H_2O; cerium oxide (IV)-20-gadolinium doped nanopowder, and cerium oxide (IV)-15-samaria doped nanopowder, exhibited.

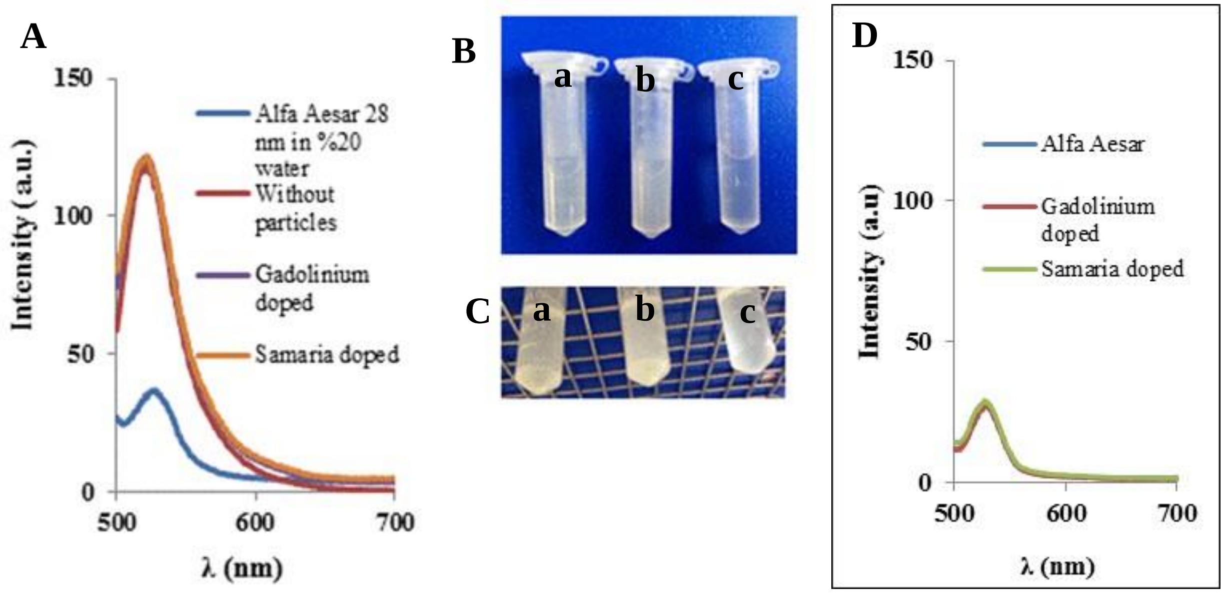

Figure S5. A) Fluorescence spectra of 1.05 nM FAM-ssDNA fluorophore before (red line) and after exposure to ceria particles (9.67 mg/L). **B)** Pictures of different types of nCe particles a) Gadolinium doped b) Samaria doped c) Alfa Aesar 28 nm in %20 water **C)** Corresponding pictures of particles after 5 min incubation. Alfa Aesar particles stay well dispersed while the larger particles formed aggregates. **D)** Fluorescence spectra of particles alone.

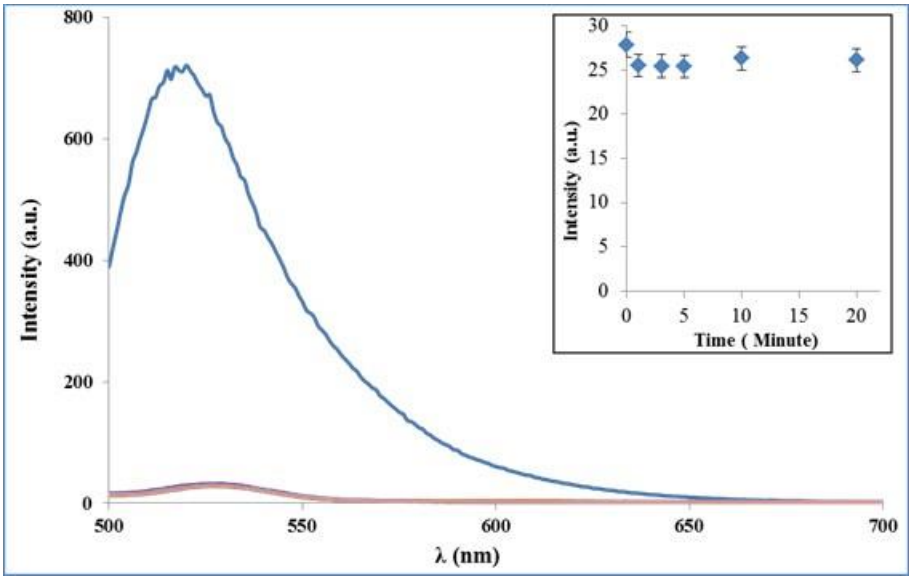

Figure S6. Fluorescence quenching of FAM-labeled ssDNA by nanoceria (2.2 mg/L) as a function of time.

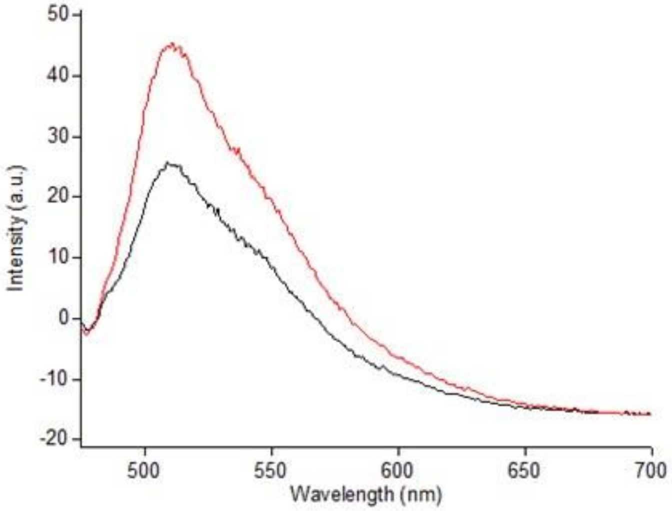

Figure S7. Fluorescence quenching of rhodamine 6G by nanoceria (0.5 mg/L); red line spectra, only dye; black line spectra, dye in the presence of nanoceria.

Table S2. Summary of detection principle and performance of the NanoCeracQ assay described in this work, presented comparatively with other nanoparticle based fluorescence quenchers reported in literature.

Nanoparticle Quencher	*Detected molecule*	*Detection Principle*	*Detection Limit nM*	*Linear Range nM*	*Refs.*
Gold	Antigen	Gold nanoparticles coated with monoclonal antibodies are used for fluorescence quenching of fluorescein isothiocyanate.	0.17	15-400	1
Gold	Target DNA	Fluorescence quenching by gold nanoparticles employed for immobilization of capture sequences and as nanoquenchers of fluorophores.	2	1.4 - 92	2
Silica	2,4,6-trinitrotoluene (TNT)	Fluorescent silica particles can specifically bind TNT species by formation of charge-transfer complexes. Formed TNT-amine complexes bound at the silica surface suppresses the fluorescence emission of the dye.	1	-	3
Titanium Oxide	Dopamine, levodopa, adrenaline, catechol	Suppression of fluorescence signal of fluorescein at 520 nm due to fluorescence quenching between the complexes of titania /enediol ligands and fluorescein.	33.5, 81.8, 20.3, 92.1	500-100000 500-100000 1000-50000 1000-50000	4
Graphene	Thrombin	Fluorescence quenching of FAM-aptamer by graphene. The fluorescence recovery is subsequently induced by formation of quadruplex-thrombin complexes	0.031	0.0625-0.1875	5
Graphene Oxide	Target DNA	Positively charged dye interacts with negatively charged graphene oxide to form a fluorescence-quenched charge-transfer complex. Fluorescence is recovered by addition of the target DNA.	1	-	6
Graphene Oxide	Hg^{2+}	In the absence of Hg^{2+}, DNA and probes are adsorbed to graphene oxide and the fluorescence of the probe is quenched. In the presence of Hg^{2+} dsDNA is released and the fluorescence is recovered.	0.3	0-1	7
Nanoceria	Target DNA	The strong binding affinity of nanoceria for the FAM-ssDNA is quenching the fluorescence of the FAM label, suppressing the fluorescent signal. In the presence of target, formation of dsDNA duplexes upon hybridization induces desorption of the probe from the particle surface decreasing the signal.	0.12	1.1 - 37	***This work***

References

1 Ao, L., Gao, F., Pan, B., He, R. & Cui, D. Fluoroimmunoassay for antigen based on fluorescence quenching signal of gold nanoparticles. *Analytical chemistry* **78**, 1104-1106 (2006).

2 Wu, Z.-S., Jiang, J.-H., Fu, L., Shen, G.-L. & Yu, R.-Q. Optical detection of DNA hybridization based on fluorescence quenching of tagged oligonucleotide probes by gold nanoparticles. *Analytical biochemistry* **353**, 22-29 (2006).

3 Gao, D. *et al.* Resonance energy transfer-amplifying fluorescence quenching at the surface of silica nanoparticles toward ultrasensitive detection of TNT. *Analytical chemistry* **80**, 8545-8553 (2008).

4 Wu, H.-P., Cheng, T.-L. & Tseng, W.-L. Phosphate-modified TiO2 nanoparticles for selective detection of dopamine, levodopa, adrenaline, and catechol based on fluorescence quenching. *Langmuir* **23**, 7880-7885 (2007).

5 Chang, H., Tang, L., Wang, Y., Jiang, J. & Li, J. Graphene fluorescence resonance energy transfer aptasensor for the thrombin detection. *Analytical chemistry* **82**, 2341-2346 (2010).

6 Balapanuru, J. *et al.* A Graphene Oxide–Organic Dye Ionic Complex with DNA-Sensing and Optical-Limiting Properties. *Angewandte Chemie* **122**, 6699-6703 (2010).

7 Huang, J., Gao, X., Jia, J., Kim, J.-K. & Li, Z. Graphene Oxide-Based Amplified Fluorescent Biosensor for Hg2+ Detection through Hybridization Chain Reactions. *Analytical chemistry* **86**, 3209-3215, doi:10.1021/ac500192r (2014).
